# Effect of Nocturnal Oxygen Therapy on Daytime Pulmonary Hemodynamics in Patients With Chronic Obstructive Pulmonary Disease Traveling to Altitude: A Randomized Controlled Trial

**DOI:** 10.3389/fphys.2021.689863

**Published:** 2021-07-07

**Authors:** Mona Lichtblau, Tsogyal D. Latshang, Sayaka S. Aeschbacher, Fabienne Huber, Philipp M. Scheiwiller, Stefanie Ulrich, Simon R. Schneider, Elisabeth D. Hasler, Michael Furian, Konrad E. Bloch, Stéphanie Saxer, Silvia Ulrich

**Affiliations:** Department of Pulmonology, University Hospital Zurich, Zurich, Switzerland

**Keywords:** chronic obstructive pulmonary disease, altitude, oxygen, echocardiography, right heart function

## Abstract

**Introduction:**

We investigated whether nocturnal oxygen therapy (NOT) mitigates the increase of pulmonary artery pressure in patients during daytime with chronic obstructive pulmonary disease (COPD) traveling to altitude.

**Methods:**

Patients with COPD living below 800 m underwent examinations at 490 m and during two sojourns at 2,048 m (with a washout period of 2 weeks < 800 m between altitude sojourns). During nights at altitude, patients received either NOT (3 L/min) or placebo (ambient air 3 L/min) via nasal cannula according to a randomized crossover design. The main outcomes were the tricuspid regurgitation pressure gradient (TRPG) measured by echocardiography on the second day at altitude (under ambient air) and various other echocardiographic measures of the right and left heart function. Patients fulfilling predefined safety criteria were withdrawn from the study.

**Results:**

Twenty-three COPD patients [70% Global Initiative for Chronic Obstructive Lung Disease (GOLD) II/30% GOLD III, mean ± SD age 66 ± 5 years, FEV_1_ 54% ± 13% predicted] were included in the per-protocol analysis. TRPG significantly increased when patients traveled to altitude (from low altitude 21.7 ± 5.2 mmHg to 2,048 m placebo 27.4 ± 7.3 mmHg and 2,048 m NOT 27.8 ± 8.3 mmHg) difference between interventions (mean difference 0.4 mmHg, 95% CI −2.1 to 3.0, *p* = 0.736). The tricuspid annular plane systolic excursion was significantly higher after NOT vs. placebo [2.6 ± 0.6 vs. 2.3 ± 0.4 cm, mean difference (95% confidence interval) 0.3 (0.1 − 0.5) cm, *p* = 0.005]. During visits to 2,048 m until 24 h after descent, eight patients (26%) using placebo and one (4%) using NOT had to be withdrawn because of altitude-related adverse health effects (*p* < 0.001).

**Conclusion:**

In lowlanders with COPD remaining free of clinically relevant altitude-related adverse health effects, changes in daytime pulmonary hemodynamics during a stay at high altitude were trivial and not modified by NOT.

**Clinical Trial Registration:**

www.ClinicalTrials.gov, identifier NCT02150590.

## Introduction

Chronic obstructive pulmonary disease (COPD) is a highly prevalent disease and according to the World Health Organization one of the three leading causes of death worldwide (Rhodes et al., 1991). Inhaled noxious particles, mainly cigarette smoke and indoor air pollution, cause chronic inflammation leading to increased collapsibility and narrowing of the airways reflected by a decreased forced expiratory volume in 1 s (FEV_1_) (Global Initiative for Chronic Obstructive Lung Disease, 2019). It is known that patients with COPD frequently suffer from right heart dysfunction due to progressive pulmonary hypertension (PH) especially during exercise and in the presence of frequent comorbidities, such as obstructive sleep apnea or nocturnal hypoxemia (Macnee, 2010; Ulrich et al., 2010; Fossati et al., 2014; Global Initiative for Chronic Obstructive Lung Disease, 2019). It has been shown that COPD patients with an elevated pulmonary artery pressure (PAP) have a worse survival than those with a normal PAP (Macnee, 2010).

The current Global Initiative for Chronic Obstructive Lung Disease (GOLD) guidelines include recommendations for the use of long-term oxygen therapy (>15 h/day) in hypoxemic patients as well as the use of short-term oxygen therapy during air travel (Johnson, 2010). During flights, a partial pressure of oxygen (PaO_2_) of at least 50 mmHg should be maintained, and patients with an oxygen saturation (SpO_2_) above 95% at rest and SpO_2_ > 84% during the 6-min walking test at sea level are usually able to travel without supplemental oxygen. Recommendations for altitude sojourns in COPD patients are lacking; meanwhile, several studies on COPD patients and altitude travel have been conducted (Furian et al.,2018b,c, 2019; Muralt et al., 2018; Latshang et al., 2019; Lichtblau et al.,2019a,b, 2020; Schwarz et al., 2019; Tan et al., 2020; Carta et al., 2021). It is known that patients with COPD may suffer from hypoxemia when traveling to altitude and that altitude travel increases PAP, induces the occurrence of intracardiac and intrapulmonary right-to-left shunts, and reduces exercise capacity (Furian et al.,2018a,b; Lichtblau et al., 2018, 2019b, 2020; Latshang et al., 2019; Schwarz et al., 2019).

Nocturnal oxygen therapy (NOT) has been shown to improve hypoxemia, sleep apnea, and subjective sleep quality in patients with COPD traveling to altitude (Tan et al., 2020). Whether NOT positively affects the altitude-induced increase of the PAP is unknown. Thus, the aim of the current study was to investigate whether NOT could mitigate the effect of altitude on PAP in stable COPD patients traveling to 2,048 m.

## Materials and Methods

### Study Design

This randomized, placebo-controlled, crossover trial in patients with COPD living below 800 m was performed between January 1 and October 31, 2014 within the scope of a project evaluating effects of oxygen on various outcomes in COPD patients staying at high altitude. Baseline characteristics and data on nocturnal oxygenation, sleep apnea, and altitude-related adverse health effects have been reported (Tan et al., 2020); but data on echocardiographic findings, the focus of the current study, have not been published. Patients were assessed at low altitude (Zurich, 490 m); and during two altitude sojourns for 2 days at 2,048 m (St. Moritz), where they either received NOT or placebo (ambient air) during nights, in a randomized order, echocardiography was performed in the morning after the first night at altitude. Between the altitude sojourns, they had a 2-weeks washout period < 800 m. The protocol was approved by the cantonal ethics committee of Zurich (KEK 2013–0088), and all patients gave written informed consent.

### Patients

Adult patients (18–75 years) diagnosed with COPD GOLD grades 2–3 permanently living < 800 m were included. Exclusion criteria were hypoxemia defined as oxygen saturation by pulse oximetry (SpO_2_) lower than 92% at 490 m, home oxygen or continuous positive airway pressure (CPAP) therapy, any uncontrolled cardiovascular disease, or history of obstructive sleep syndrome (Tan et al., 2020).

### Randomization, Intervention, Blinding, and Sample Size

Patients were randomly assigned by blocks of four: (1) 490 m, placebo 2,048 m, NOT 2,048 m; (2) 490 m, NOT 2,048 m, placebo 2,048 m; (3) placebo 2,048 m, NOT 2,048 m, 490 m; and (4) NOT 2,048 m, placebo 2,048 m, 490 m. Patients traveled from 490 to 2,048 m by train and car within 3 h. During nights at altitude, patients received either 3 L/min NOT or placebo (ambient air) provided by an identical-looking oxygen concentrator (EverFlo, Philips Respironics, Zofingen, Switzerland) via nasal cannula. Patients were monitored with an online sleep study with camera view during the night, ensuring compliance. Patients and assessors of echocardiography were blinded to the gas mixture.

A total of 18 participants were required for a minimal detectable change of tricuspid regurgitation pressure gradient (TRPG) of 5 (SD ± 5 mmHg) with a power of 0.8 and a significance level of 0.05.

### Assessments

Echocardiography was performed at 490 m and on the second day at 2,048 m (after the first night with NOT/placebo) during ambient air breathing as described before (Lichtblau et al., 2019b). Echocardiographic recordings were performed with a real-time, phased array sector scanner (CX 50, Philips, Philips Respironics, Zofingen, Switzerland) with an integrated color Doppler system and a transducer containing crystal sets for imaging (1–5 MHz) and for continuous-wave Doppler. Measurements were carried out according to guidelines of the European Association of Echocardiography (Galderisi et al., 2017). Systolic PAP (sPAP) was calculated by summing up the TRPG (calculated with the Bernoulli equation from the peak velocity of the tricuspid regurgitation ΔP = 4 × V^2^_m__ax_) and the right atrial pressure (RAP; estimated on the basis of the inferior vena cava diameter and collapse) (Rudski et al., 2010; Lichtblau et al., 2019b). The right atrium and ventricle were manually traced, and fractional area change (FAC) calculated (% systolic/diastolic right ventricular area). Tricuspid annular plane systolic excursion (TAPSE) was measured by M-mode and the right ventricular free wall velocity by tissue Doppler imaging. Mean PAP (mPAP) was calculated from sPAP with the following formula: mPAP = sPAP × 0.61 + 2 (Chemla et al., 2004). Pulmonary artery wedge pressure (PAWP) was calculated as 1.24 × (E/e′) + 1.9 (Doutreleau et al., 2016). Cardiac output was calculated using the left ventricular (LV) outflow tract velocity time integral. Pulmonary vascular resistance (PVR) was calculated with the following formula: PVR = (mPAP − PAWP)/cardiac output, and left heart function was assessed according to the current guidelines (Galderisi et al., 2017). Pressure/flow relation was calculated with mPAP/cardiac output, and right ventricular arterial coupling was calculated with TAPSE/sPAP (Tello et al., 2019).

Arterial blood gas analysis was performed on radial artery blood sample and was drawn in the morning during ambient air breathing (RapidPoint 405, Siemens Healthcare Diagnostics AG, Zurich, Switzerland).

### Outcomes

Primary outcome was the differences in TRPG between NOT and placebo treatment. Secondary outcomes were additional echocardiographic variables of the right and left heart.

Patients who suffered from an altitude-related adverse health effects were excluded from the study, according to the following predefined safety criteria (Tan et al., 2020):

– severe hypoxemia (SpO_2_ < 75% for > 30 min at any time while at high altitude),
– intercurrent illness (e.g., exacerbation of COPD, cardiovascular disease, infection, or new diseases),
– AMS (Environmental Symptoms Questionnaire Cerebral score ≥ 0.7; Sampson et al., 1983), and
– any other conditions that required therapy.

### Statistical Analysis

Analysis was performed per protocol including patients who underwent all three sessions; missing values were not replaced. The data are summarized as mean and SD. Mean differences and 95% confidence intervals (95% CIs) between measures at 490 m and at 2,048 m with NOT or placebo were calculated. All outcomes were computed using linear mixed models with outcomes as dependent variables and altitude and intervention as independent variables. We herewith included all time points in one model and assumed that data are normally distributed (*n* > 30). Statistical significance was assumed when 95% CIs of mean differences did not overlap zero or *p* < 0.05. Statistical analysis was performed with Stata version 15.1 (StataCorp, College Station, TX, United States).

## Results

**FIGURE 1:**
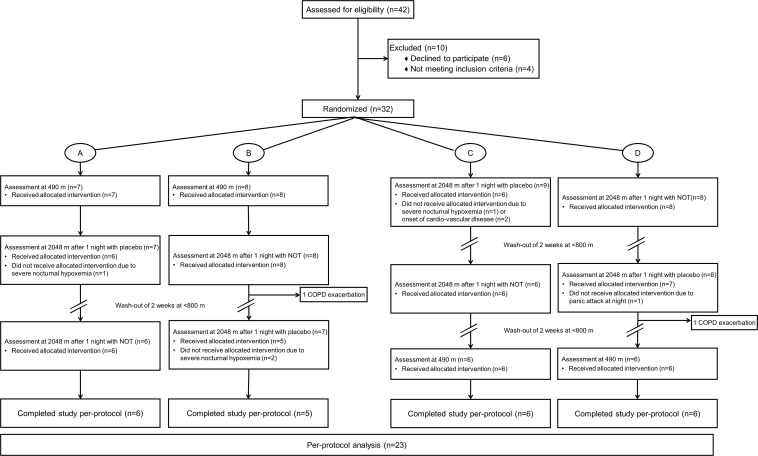
Patient flow.

The patient flow is shown in Figure 1. Five patients were excluded from the analysis because they received oxygen therapy at night in the placebo phase due to hypoxemia during sleep, according to predefined safety criteria. Two patients dropped out due to COPD exacerbations, and another two patients due to acute onset of cardiovascular disease. A total of 23 patients were included in the per-protocol analysis.

Patients were on average 66 ± 5 years old (10 males and 13 females, FEV_1_ 53.7% ± 12.8% predicted), and baseline characteristics including medications are listed in Table 1. Patients were mostly classified as COPD GOLD II (70%) and the remaining as GOLD III.

**TABLE 1:** Baseline characteristics.

Age, years	66.0 ± 5.1
Sex, males/females	10/13
Body mass index, kg/m^2^	25.3 ± 3.9
Pack years, years	38.2 ± 31.5
COPD GOLD	
II	16 (70)
III	7 (30)
FEV_1_,% predicted	53.7 ± 12.8
FVC,% predicted	90.3 ± 11.5
FEV_1_/FVC	54.3 ± 11.8
TLco adjusted for hemoglobin,% predicted	71.4 ± 20.2
6-min walking distance, m	543 ± 89
SpO_2_ at end of 6-min walking distance,%	92.5 ± 3.9
Medication	
Inhaled glucocorticosteroids	2 (9)
Inhaled β-adrenergics	17 (74)
Inhaled anticholinergics	18 (78)
Diuretics	2 (9)
Antihypertensive medication	12 (52)
Antidiabetics	3 (13)
Antidepressants	3 (13)

*Values are presented as mean ± SD or numbers and proportions.*

*COPD, chronic obstructive pulmonary disease; GOLD, Global Initiative for Chronic Obstructive Lung Disease; FEV_1_, forced expiratory volume in the first second of expiration; FVC, forced vital capacity; TLco, diffusing capacity for carbon monoxide; SpO_2_, oxygen saturation.*

At 2,048 m, patients revealed a significantly higher heart rate (72 ± 9 bpm at low altitude vs. 79 ± 9 bpm at 2,048 m placebo and 78 ± 11 bpm at 2,048 m NOT), along with a significant lower oxygen saturation (95.3% ± 1.6% vs. 90.6% ± 2.8% with placebo and 91.3% ± 3.3% with NOT) and a lower PaO_2_ (67.7 ± 6.6 vs. 59.3 ± 6.0 mmHg with placebo and 58.7 ± 7.2 mmHg with NOT) and PaCO_2_ (36.9 ± 3.0 vs. 34.2 ± 3.8 mmHg with placebo, 34.1 ± 4.1 mmHg with NOT) compared with low altitude (Table 2). Hematocrit did significantly differ from 490 to 2,048 m with NOT (41.2% ± 3.2% vs. 42.5% ± 3.6% with placebo, *p* = 0.078 and 42.9% ± 3.9% with NOT, *p* = 0.037) and hemoglobin with both placebo and NOT (14.1 ± 1.2 vs. 14.5 ± 1.2 g/L, *p* = 0.031 and 14.6 ± 1.3 g/L with NOT, *p* = 0.016) (Table 2). No relevant differences between placebo and NOT treatment were found.

**TABLE 2:** Vital signs and blood gases.

	490 m baseline	2,048 m placebo	2,048 m NOT	2,048 m NOT vs. placebo	*p*-value
Heart rate, bpm	72 ± 9	79 ± 9^a^	78 ± 11^a^	−1 (−4 to 2)	0.544
Diastolic blood pressure, mmHg	73 ± 10	78 ± 10	78 ± 9	1(−3 to 5)	0.697
Systolic blood pressure, mmHg	129 ± 19	133 ± 14	130 ± 19	−3 (−9 to 2)	0.252
SpO_2_,%	95.3 ± 1.6	90.6 ± 2.8^a^	91.3 ± 3.3^a^	0.7 (−0.5 to 1.9)	0.250
PaO_2_, mmHg	67.7 ± 6.6	59.3 ± 6.0^a^	58.7 ± 7.2^a^	−0.6 (−2.6 to 1.3)	0.519
PaCO_2_, mmHg	36.9 ± 3.0	34.2 ± 3.8^a^	34.1 ± 4.1^a^	0.2 (−1.1 to 1.5)	0.758
Hematocrit,%	41.2 ± 3.2	42.5 ± 3.6	42.9 ± 3.9^a^	0.1 (−0.5 to 0.8)	0.708
Hemoglobin, g/dl	14.1 ± 1.2	14.5 ± 1.2^a^	14.6 ± 1.3^a^	0.0 (−0.2 to 0.3)	0.761

*Data are presented as mean ± SD.*

*SpO_2_, oxygen saturation by pulse oximetry; PaO_2_, partial pressure of arterial oxygen; PaCO_2_, partial pressure of arterial carbon dioxide.*

*^*a*^p < 0.05 vs. 490 m.*

**FIGURE 2:**
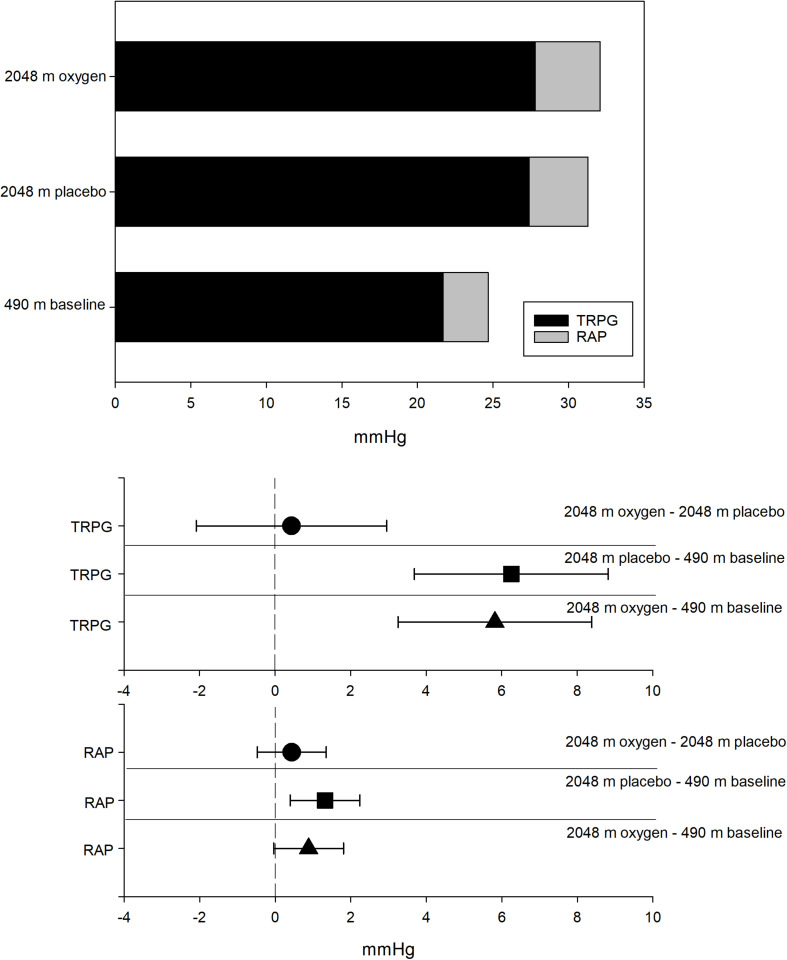
Systolic pulmonary artery pressure displayed as sum of the tricuspid regurgitation pressure gradient (TRPG) and the right atrial pressure (RAP) at 490 and 2,048 m [with placebo and nocturnal oxygen therapy (NOT), respectively]. The lower part shows the differences of the TRPG and the RAP for ●, 2,048 m oxygen, 2,048 m placebo; ■, 2,048 m placebo, 490 m baseline; and ▲, 2,048 m oxygen, 490 m baseline.

Table 3 displays echocardiography measurements of the participants at low altitude and at high altitude with and without NOT. COPD patients significantly increased their TRPG from 490 to 2,048 m (490 m 21.7 ± 5.2 mmHg vs. 2,048 m 27.4 ± 7.3 mmHg with placebo and 2,048 m 27.8 ± 8.3 mmHg with NOT) and consequently the sPAP and mPAP with no significant difference between treatments (Figure 2).

**TABLE 3:** Echocardiographic measurements.

	490 m baseline	2,048 m placebo	2,048 m NOT	2,048 m NOT vs. placebo	*p*-value
TRPG, mmHg	21.7 ± 5.2	27.4 ± 7.3^a^	27.8 ± 8.3^a^	0.4 (−2.1 to 3.0)	0.736
sPAP, mmHg	24.7 ± 5.2	31.3 ± 7.2^a^	32.1 ± 8.9^a^	0.9 (−2.1 to 3.9)	0.517
mPAP, mmHg	16.4 ± 4.4	21.1 ± 4.4^a^	21.6 ± 5.4^a^	0.5 (−1.3 to 2.4)	0.560
RAP, mmHg	3.0 ± 0.0	3.9 ± 1.9	4.3 ± 2.2^a^	0.4 (−0.5 to 1.4)	0.350
RA area, cm^2^	12.6 ± 2.3	12.9 ± 3.5	13.8 ± 4.2^a^	0.9 (−0.2 to 2.0)	0.123
RVEDA, cm^2^	14.4 ± 4.0	13.5 ± 3.8	13.2 ± 3.5	−0.3 (−1.6 to 1.0)	0.682
RVESA, cm^2^	8.6 ± 3.0	8.3 ± 2.8	8.2 ± 2.9	−0.1 (−1.0 to 0.8)	0.809
FAC,%	41.0 ± 8.7	38.6 ± 6.6	38.0 ± 10.2	−0.7 (−5.2 to 3.9)	0.772
TAPSE, cm	2.5 ± 0.5	2.3 ± 0.4	2.6 ± 0.6	0.3 (0.1 to 0.5)	0.005
Eccentricity index end-diastolic	1.1 ± 0.1	1.1 ± 0.1	1.1 ± 0.1	0.0 (−0.0 to 0.1)	0.288
Eccentricity index end-systolic	1.0 ± 0.1	1.1 ± 0.1	1.1 ± 0.1^a^	0.0 (−0.0 to 0.7)	0.293
RV anterior wall diameter, cm	0.6 ± 0.2	0.5 ± 0.1	0.6 ± 0.3	0.1 (0.0 to 0.2)^b^	0.046
RV diameter end-diastolic, cm	2.9 ± 0.5	2.7 ± 0.7	2.8 ± 0.5	0.2 (−0.1 to 0.4)	0.317
TDI tricuspid annular systolic velocity, cm/s	14.6 ± 2.9	15.9 ± 2.9	16.7 ± 4.5^a^	0.9 (−0.6 to 2.3)	0.237
Stroke volume, ml	82.4 ± 18.0	83.6 ± 18.0	90.6 ± 22.7	6.8 (−3.4 to 16.9)	0.194
Cardiac output, L/min	6.0 ± 1.6	6.5 ± 1.6	6.9 ± 1.7^a^	0.4 (−0.4 to 1.2)	0.357
Cardiac index, L/min/m^2^	3.4 ± 0.9	3.6 ± 0.8	3.9 ± 0.9^a^	0.2 (−0.2 to 0.7)	0.329
mPAP/cardiac output	3.0 ± 0.9	3.5 ± 1.1^a^	3.4 ± 1.1	−0.1 (−0.6 to 0.4)	0.764
TAPSE/sPAP, mm/mmHg	1.1 ± 0.3	0.8 ± 0.2^a^	0.8 ± 0.2^a^	0.1 (−0.0 to 0.2)	0.209
PAWP, mmHg	10.3 ± 2.3	9.0 ± 2.2	10.9 ± 4.5	1.9 (0.4 to 3.4)	0.011
PVR, WU	1.2 ± 0.6	2.1 ± 1.1^a^	1.8 ± 1.1^a^	−0.3 (−0.8 to 0.1)	0.134
PVR corrected for hematocrit	1.4 ± 0.8	2.3 ± 1.2^a^	2.2 ± 1.9^a^	−0.1 (−0.8 to 0.6)	0.785
Ejection fraction (biplan),%	63.5 ± 6.9	67.1 ± 6.5^a^	66.5 ± 7.3	−0.5 (−3.8 to 2.7)	0.750
LV internal dimension end-diastolic, cm	4.2 ± 0.9	4.1 ± 0.6	4.1 ± 0.4	−0.0 (−0.3 to 0.3)	0.969
LV internal dimension end-systolic, cm	2.8 ± 0.6	2.7 ± 0.5	2.8 ± 0.5	0.2 (−0.1 to 0.4)	0.249
LV posterior wall end-diastolic, cm	1.0 ± 0.6	0.8 ± 0.1^a^	0.9 ± 0.1	0.0 (−0.1 to 0.2)	0.633
MV E/A	1.0 ± 0.4	0.8 ± 0.3^a^	0.9 ± 0.3^a^	0.1 (−0.0 to 0.2)	0.189

*Data are presented as mean ± SD.*

*TRPG, tricuspid regurgitation pressure gradient; sPAP, systolic pulmonary artery pressure; mPAP, mean pulmonary artery pressure; PAWP, pulmonary arterial wedge pressure; RAP, right atrial pressure; RA, right atrium; RVEDA, right ventricle end-diastolic area; RVESA, right ventricle end-systolic area; FAC, fractional area change; TAPSE, tricuspid annular plane systolic excursion; RV, right ventricle; TDI, tissue Doppler imaging; PVR, pulmonary vascular resistance; LV, left ventricle; MV, mitral valve; Peak E, early mitral inflow velocity; Peak A, peak atrial filling velocity; E/A, ratio of transmitral early diastolic to late diastolic velocity.*

*^*a*^p < 0.05 vs. 490 m.*

TAPSE was higher after NOT vs. placebo [2.5 ± 0.5 vs. 2.3 ± 0.4 cm with placebo and 2.6 ± 0.6 cm with NOT, mean differences (95% CI) 0.3 (0.1 −0.5), *p* = 0.005], whereas the right ventricular anterior wall diameter was measured slightly higher under NOT vs. placebo [0.6 ± 0.2 vs. 0.5 ± 0.3 cm with placebo and 0.6 ± 0.3 cm with NOT, mean difference (95% CI) 0.1 (0.0−0.2), *p* = 0.046].

Right ventricular arterial coupling was decreased and PVR elevated at 2,048 m with no difference between treatments [coupling: 490 m 1.1 ± 0.3 mm/mmHg vs. 2,048 m 0.8 ± 0.2 mm/mmHg with placebo and 0.8 ± 0.2 mm/mmHg with NOT, both *p* < 0.05; PVR: 490 m 1.2 ± 0.6 Wood units (WU) vs. 2,048 m 2.1 ± 1.1 WU with placebo and 1.8 ± 1.1 WU with NOT, both *p* < 0.05] (Table 3).

## Discussion

This is the first randomized controlled trial investigating the effect of NOT on the PAP in patients with COPD traveling to altitude. NOT did not mitigate the effect of hypoxia-induced increase of the TRPG measured on ambient air the second day in lowlanders with COPD traveling to 2,048 m compared with placebo.

At low altitude, TRPG of the presently investigated COPD patients was 21.7 ± 5.2 mmHg and thus within a normal range and comparable with that of healthy subjects, where a tricuspid regurgitation velocity of 2.55 m/s, corresponding to a TRPG of 26 mmHg, is defined as the upper limit of normal (Marra et al., 2018).

The altitude-induced increase of TRPG was less pronounced than in a study published previously (Lichtblau et al., 2019b), where COPD patients increased their TRPG from 23 (19; 29) mmHg to 32 (25; 41) mmHg during a sojourn to 2,590 m in comparison to the increase to 27.4 ± 7.3 mmHg with placebo and to 27.8 ± 8.3 mmHg NOT at an altitude of 2,048 m in the current study. The difference in altitude can be one of the reasons for this less pronounced PAP increase or the fact that in the current study patients with altitude-related adverse health effects were excluded. At an altitude of 3,100 m, even higher values were obtained (from 20 ± 4 mmHg at lowland to 31 ± 9 mmHg at highland) in COPD patients with milder disease severity (GOLD 1 and 2 only) (Lichtblau et al.,2019a,b). In another study, lifelong altitude residents with COPD showed a significantly higher mPAP than did lowlanders with COPD (lowlanders 26.8 ± 11.1 mmHg and highlanders 36.1 ± 10.2 mmHg) and also comparably higher with our results (mPAP 16 ± 4 mmHg at lowland, 21 ± 4 mmHg at altitude with placebo, and 22 ± 5 mmHg with NOT). However, while mPAP in the current study was derived from sPAP, Guvenc et al. (2013) estimated mPAP from pulmonary acceleration time, and therefore, the comparison of the studies is difficult.

Likewise to TRPG, for heart rate and FAC, similar changes in patients with COPD at increasing altitudes were found (Lichtblau et al., 2019b).

There were two significant differences between treatments, namely, the TAPSE [0.3 (0.1−0.5)] and the PAWP [1.9 (0.4−3.4)]. The difference of the PAWP is difficult to interpret since this variable is prone to measurement inaccuracy, and we therefore caution to overinterpret this result. TAPSE as a measure of right heart function was better after NOT, potentially indicating a beneficial effect of NOT or showing an increase in right heart contractility as a reaction to the acute drop in oxygen levels after the night on NOT.

Exposure to altitude induced an increase in PVR in both treatment arms, with a tendency toward a lesser increase with NOT but no relevant difference between the groups with a mean difference of −0.33 (−0.76−0.10) WU. In a previous study with 95 COPD patients exposed to 3,100 m, preventive treatment with dexamethasone showed a significant mitigating effect with a mean difference of −0.5 (−0.9 to −0.1) WU between the placebo and the treatment group (Lichtblau et al., 2019a). The less pronounced and non-significant effect of NOT in the current study might be explained by the fact, that in this study, all measurements were performed on ambient air at 2,048 m after the night with NOT; and thus, a potential vasodilator effect of oxygen may not have persisted anymore, which was already shown in the arterial blood gas analysis published in the main paper where no difference was found between NOT and placebo at 2,048 m (Tan et al., 2020). Looking more closely at the course of PAP and PVR during acute exposure to hypoxia reveals that an excess increase occurs within the acute phase (<180 min) as described *in vitro* and *in vivo*, followed by a dip and a steady increase over the next hours (Sommer et al., 2016). In the current study, echocardiography was performed only few hours after receiving either oxygen or ambient air, and therefore, patients receiving NOT were potentially still in the overcompensating phase. Two studies in patients with PH have shown reduction in mPAP during acute exposure with oxygen therapy. The first study in 104 PH patients revealed a reduction of mPAP from 46.4 ± 1.3 to 42.2. ± 1.3 mmHg with 5 L/min oxygen therapy via nasal cannula (Leuchte et al., 2013), and the second study in 28 PH patients has shown that acute hyperoxia with 100% FiO_2_ lowers mPAP by 4 (2–6) mmHg (Groth et al., 2018). Hemodynamics after oxygen withdrawal have not been studied; however, Leuchte et al. (2013) found that the effect of oxygen on invasively measured hemodynamics disappeared 15 min after stopping supplemental oxygen. We can only speculate that patients in the current study had a mitigating effect on PAP while receiving NOT during the nights; however, potential beneficial effects seem not to be sustainable during the following morning when echocardiography was performed, and whether NOT given for several nights would be beneficial during longer altitude sojourns remains unclear. At lowland, a study in COPD patients did not show a significant benefit after 2 years with NOT on PAP (control group 19.8 ± 5.6−20.5 ± 6.5 mmHg and NOT 18.3 ± 4.7 −19.5 ± 5.3 mmHg, *p* = 0.79). A recent trial was stopped prematurely because of recruitment and retention difficulties, showing—in this underpowered analysis—no clear positive or negative effect on survival or progression to long-term oxygen therapy in COPD patients (Lacasse et al., 2020) and therefore emphasizing again the need of efficiently powered trials to study the long-term effect of NOT in patients with COPD. However, according to our previous publication from the current trial, NOT had a positive effect on various sleep measures, but not on daytime exercise capacity, blood gases, or cognitive performance compared with placebo at altitude (Tan et al., 2020). Echocardiographic right-to-left shunt measurements were not performed during this study, but from previous trials, it is known that hypobaric hypoxia induces a higher prevalence of intracardiac and intrapulmonary right-to-left shunts in lowlander COPD patients traveling to altitude (Lichtblau et al., 2020); and therefore, patients with COPD and right-to-left shunt might even less profit from supplemental oxygen. On the contrary, Lovering et al. (2008) have demonstrated prevention of exercise-induced intrapulmonary shunts in healthy humans with high levels of hyperoxia (100% FiO_2_).

### Limitations

According to the safety criteria of the current study protocol, patients who experienced altitude-related adverse health effects received oxygen and were excluded from this analysis, which biased our results toward no between-group difference, as the proportion of excluded patients in the group receiving placebo was much higher than that in those who received NOT. However, performing an echocardiography immediately during the night when patients under placebo got hypoxemic was not feasible, and performing an echocardiography under oxygen therapy in these patients would also have biased the results. We chose the time point of echocardiography after the first night at altitude on NOT vs. placebo in order to see whether NOT would have a prolonged effect on pulmonary hemodynamics even after several hours on ambient air. Whether long-term treatment with oxygen at altitude or a longer treatment with NOT would have had a beneficial effect on pulmonary hemodynamics remains to be investigated. The sample size of our study was relatively small, but estimation for a minimal detectable change of a TRPG 5 ± 5 mmHg with a power of 0.8 and a significance level of 0.05 would have required 18 participants, a number that was met by the 23 patients who completed the study. We included COPD GOLD 2 and 3, and our data cannot be extrapolated for more or less severe COPD patients.

## Conclusion

In lowlanders with COPD remaining free of clinically relevant altitude-related adverse health effects, changes in daytime pulmonary hemodynamics during a stay at high altitude were trivial and not modified by NOT.

## Data Availability Statement

The raw data supporting the conclusions of this article will be made available by the authors, without undue reservation.

## Ethics Statement

The studies involving human participants were reviewed and approved by the Ethics Committee of Zurich. The patients/participants provided their written informed consent to participate in this study.

## Author Contributions

SiU was the guarantor and took responsibility for the content of the manuscript, including the data and analysis. ML, SiU, and SS contributed to acquiring, analyzing, and interpreting the data, writing and revising the article critically for important intellectual content, and providing final approval of the version to be published. SA, FH, PS, StU, SRS, EH, and MF contributed to data collection, analysis, and revising the article critically for important intellectual content. SiU, TL, and KB conceived the project and contributed to data collection, analysis and interpretation, writing the manuscript, revising the article critically for important intellectual content, and providing final approval of the version to be published. All authors were took responsibility for all aspects of the reliability and freedom from bias of the data presented and their discussed interpretation.

## Conflict of Interest

SiU reports grants from Orpha Swiss and Actelion SA, and personal fees from Actelion SA, MSD, and Orpha Swiss, outside the submitted work. The remaining authors declare that the research was conducted in the absence of any commercial or financial relationships that could be construed as a potential conflict of interest.
